# Swine Inflammation and Necrosis Syndrome (SINS)

**DOI:** 10.3390/ani11061670

**Published:** 2021-06-03

**Authors:** Gerald Reiner, Josef Kuehling, Frederik Loewenstein, Mirjam Lechner, Sabrina Becker

**Affiliations:** 1 Department of Veterinary Clinical Sciences, Clinic for Swine, Justus Liebig University Giessen, Frankfurter Strasse 112, 35392 Giessen, Germany; josef.kuehling@vetmed.uni-giessen.de (J.K.); sabrina.becker-2@vetmed.uni-giessen.de (S.B.); 2 LSZ Boxberg, Seehöfer Str. 50, 97944 Boxberg, Germany; Frederik.Loewenstein@lsz.bwl.de; 3 UEG Hohenlohe, Am Wasen 20, 91567 Herrieden, Germany; mirjam.lechner@web.de

**Keywords:** tail biting, inflammation and necrosis, swine, animal welfare

## Abstract

**Simple Summary:**

Tail biting in pigs is an expression of suboptimal animal welfare. The search for causes and solutions is difficult. Recent studies have shown that injuries to the tail occur with considerable frequency, even without the intervention of other pigs, and that the injuries are not confined to the tail but can also be present in ears, teats, claws, coronary bands, heels, soles, and other body parts. This review summarizes the existing findings on a new syndrome introduced as swine inflammation and necrosis syndrome (SINS). This paper will present clinical alterations and gather evidence to better understand the underlying mechanisms. It concludes by presenting methods to combat the syndrome through improving pig husbandry and feeding and by selecting less-susceptible breeding animals.

**Abstract:**

Tail biting is a prevalent and undesirable behaviour in pigs and a major source of significant reduction in well-being. However, focusing on biting considers only one part of the solution, because tail damage can be found with a high prevalence without any action by other pigs. The lesions are not limited to the tail but can also be found in the ears, heels, soles, claw coronary bands, teats, navel, vulva, and face. Environmental improvement alone often fails to overcome the problem. This review addresses a new inflammation and necrosis syndrome in swine (SINS). It shows the clinical signs and the frequencies of occurrence in different age groups. It compiles scientific evidence from clinical and histopathological studies in newborn piglets that argue for a primary endogenous aetiology of the disease. Bringing together the findings of a broad body of research, the possible mechanisms leading to the disease are identified and then discussed. This part will especially focus on microbe-associated molecular patterns in the circulation and their role in activating defence mechanisms and inflammation. Finally, the methods are identified to ameliorate the problem by optimizing husbandry and selecting a suitable breeding stock.

## 1. Introduction

Inflammation and loss of tail integrity can seriously impair animal welfare in pigs (European Food Safety Authority (EFSA) [1,2]. One of the most studied causes is tail biting, a very prevalent undesirable behaviour that has particularly been identified in growing pigs [3,4,5,6,7].

A number of external factors are well-accepted triggers for the problem, including: insufficient activity and boredom due to poor environmental enrichment and comfort; failure to satisfy natural behaviours; stress of any kind; low-quality air, food, and water; too much sunlight; excessive temperature; regrouping; excessive housing density; inappropriate pen structure, leading to confrontation between animals or the inability of all animals to feed simultaneously; disease; malnutrition; stimuli such as blood; and many more [7,8,9,10]. Internal factors such as aggressiveness, frustration, and genetic causes have also been identified as triggers for tail biting, and the multifactorial nature of the problem cannot be overcome by focusing on individual components [7,9]. Even with intensive use of the available measures, 25–70% of animals may have damaged tails (e.g., [11,12,13]). While earlier studies focused on tail-biting as a behavioural disorder attributable to the barren environment and housing conditions, a prevalence of tail biting between 14% and 20% was recorded in 2004 under extensive outdoor conditions, as is common practice in Switzerland [14].

Tail lesions are of special interest, not only due to their severe direct impact on animal welfare but, also, because tail docking is still used as the major preventive measure in most countries [15]. Tail docking, however, further increases damage, pain, and animal welfare concerns, without completely eliminating the problem and leaving the underlying causes unresolved [16]. Despite rising demands to ban tail docking in the EU (EU directive 2008/120IEC), field observations and scientific studies have shown that discontinuing tail docking under current practical conditions can seriously increase the prevalence of tail lesions [12,17].

Evidence from research and practice suggests that tail lesions might be caused not only by tail biting but, also, by inflammation and necrosis, which can occur without any action from other pigs [18,19,20,21,22,23,24,25,26]. In 50% of litters, up to 75% of piglets [19] can be affected. These lesions are also not limited to the tail but can be observed in the ears, heels and soles, claw coronary bands, teats, navel, vulva, and face [21,22,23,24,25,26]. Most affected piglets have lesions in more than one body part. The syndrome-like combination of different body parts and the clinical domination of inflammation and necrosis in these areas led to the term swine inflammation and necrosis syndrome (SINS; [22]).

The simultaneous occurrence in such disparate body regions as tail, teats, claws and others [22,24]; the evidence that SINS can be triggered before birth, when biting and mechanical irritation (e.g., from the floor) are excluded [25]; and the histopathological evidence for vascular-associated inflammation in neonatal piglets with (still) intact epidermis [23,24] all argue for a primary endogenous cause of the syndrome.

There is much to suggest that inflammation and lesions strongly affect animal welfare in swine, that they affect much more than just the tail of the animals and that tail biting and mechanical irritation due to technopathies alone are not sufficient to comprehensively address and combat the issue. The aim of this review is to summarize the current knowledge on SINS, to offer a hypothesis and mechanisms on the development of the syndrome and, derived from these, starting points to overcome the disease.

## 2. Inflammation and Necrosis Syndrome in Swine (SINS)

**Figure 1:**
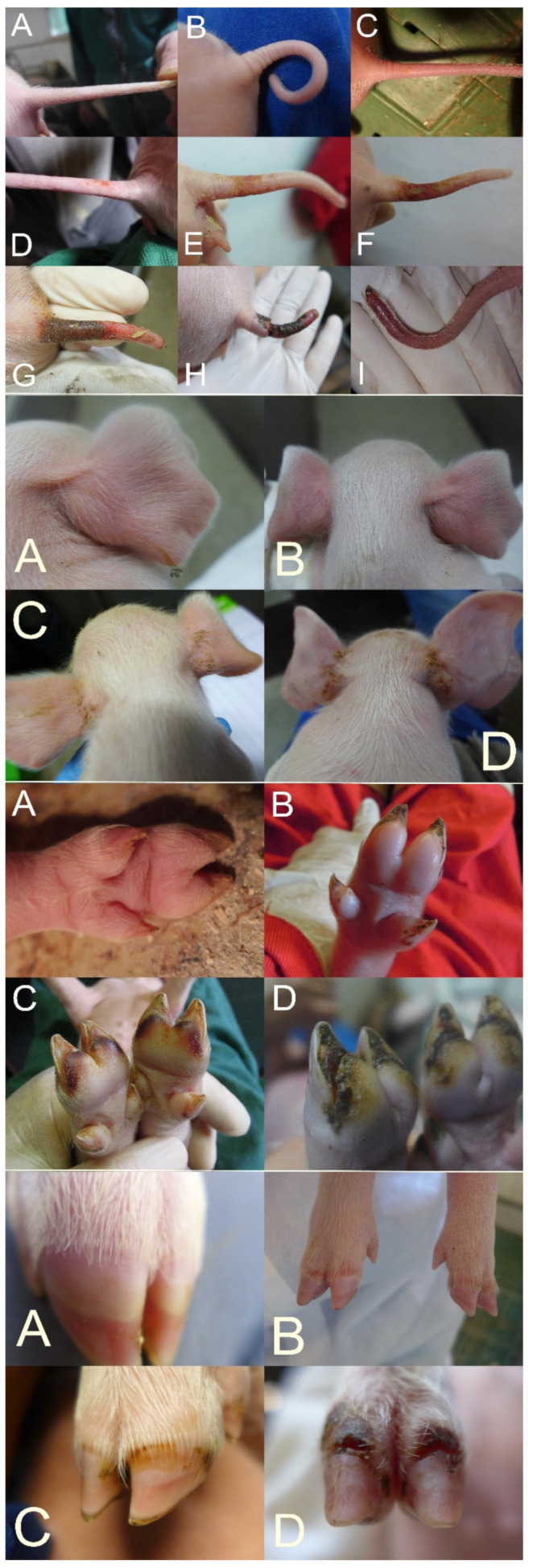
This figure shows, arranged one below the other and separated by yellow lines, different degrees of inflammation and necrosis of the tail (top, (**A**–**I**)), ears (middle top, (**A**–**D**)), claws, heels and soles (middle bottom, (**A**–**D**)), and coronary bands (bottom, (**A**–**D**)) in three-day-old piglets. Photos marked A show the physiological state without SINS. Inflammation and necrosis of the tail: (**A**) Intact tail; no swelling, no redness, no deposits or exudation and full bristles. (**B**) First stage of change: bristle loss and swelling. (**C**) Redness is added. (**D**–**G**) Various degrees of tail base inflammation and necrosis; in (**D**), the epidermis is still intact. (**H**) Severe necrosis. (**I**) Tail tip necrosis. Inflammation and necrosis of the base of the ear: (**A**) Intact ear. (**B**) Reddening of the ears. (**C,D**) Different degrees of exudative inflammation. Inflammation and necrosis of the soles and heels: (**A**) Intact foot. (**B**) Swelling. (**C**) Bleeding into the heels (also, the dew claws are affected). (**D**) Separation of sole and heels. Inflammation and necrosis in the area of the coronary bands: (**A**) Intact coronary band. **(B**) Redness. (**C**) Inflammation. (**D**) Necrosis. All photos by Mirjam Lechner.

SINS is a newly identified, distinct syndrome resulting from the combined presence and signs of clinical inflammation and dead tissue in the acral areas. It particularly affects the tail base, tail tip, ears, coronary bands, heels, soles, claw walls, teats, navel, and face (Figure 1) and can be observed in suckling piglets, weaners, and finishing pigs [21,22,23,24,25].

The signs generally start with a loss of bristles, followed by swelling and redness. At later stages, exudation and, finally, necrosis can be detected. Several studies found a loss of bristles in 30–90% of the piglets, mainly at the tail base and ears. Swelling and redness were reported at a slightly lower prevalence in the tail base, tail, and teats. Exudation and necrosis were rare in newborn piglets, with 0.7% of piglets being affected at the tail, ear, and teats [24], rising to 2–10% in three-day old piglets [23,25]. The number of affected individuals increased from suckling piglets to weaners and decreased again in fatteners but was still present at up to 6.8% (loss of bristles), 10.7% (swelling), and 10.7% (necrosis) in the tail, ear, and teats in fatteners. The claw walls, soles, and heels were also affected. Other clinical signs of the presence of local inflammation were vein combustion on the ears, teats, and veins of the hind limbs. The relationship between the total SINS score and the first appearance of signs in different body parts was shown by the authors of [25]. According to them, the first signs that can be found are a loss of bristles at the tail base and ears, redness of heels, swelling of tail base, tail, teats, and coronary band inflammation. Bleeding, exudation, necrosis, and ring tail at the tail tip were the clinical signs that developed last and only in severe cases of SINS. This differentiation is important for the early diagnosis of SINS [23]. However, the mild signs are easier to overlook.

## 3. SINS Diagnostics and Differential Diagnostics

SINS signs are relatively easy to clinically diagnose. It is important to be aware of the meaning of the clinical signs and to clean the animals, e.g., in the area of the claws, in such a way that alterations can be detected. It is also important for scoring to be conducted by an experienced person and to expect variations between the results of different observers. However, no statements are available yet on the magnitude of the deviations that should be expected.

In SINS scoring, the base of the tail, the rest of the tail, including the tail tip, the ears, the teats, the navel, the coronary bands, the claw wall, the soles, and heels are considered [21,22,23]. The face and vulva can also be recorded. To facilitate scoring with a high repeatability of results and low stress to the piglets, the body parts to be assessed should be photographed. The actual scoring can then be done based on the photos [24,25].

Although it would be possible to score the individual body parts semi-quantitatively, binary scoring has proven to be effective so far. Here, an unaffected physiological state was scored with 0 and deviation with 1. This is simpler than semi-quantitative scoring and allows for a more comprehensible evaluation. Nevertheless, a good differentiation can be achieved by collecting the binary scores for all possible findings of the respective body parts (e.g., loss of bristles (0/1), swelling (0/1), reddening (0/1), rhagades (0/1), exudation (0/1), bleeding (0/1), necrosis (0/1), and ring-shaped constrictions (0/1)) for the tail (see below).

The tail and tail base are usually scored separately, considering the loss of bristles, swelling, reddening, rhagades, exudation, bleeding, clinical signs of necrosis, and the occurrence of ring-shaped constrictions at the tail (Figure 1A–I (top)). If, for example, all these clinical signs could be observed in the tail base, the tail base score would be 8. Ear scoring is focused on the loss of bristles, congestion of ear veins, and clinical necrosis. Necrosis can include any part of the ears and, also, the ear tips (Figure 1A–D (middle top)). Teats are scored for scab formation, swelling, reddening, clinical signs of necrosis, and the congestion of blood vessels. The navel is scored for redness and swelling. The face is scored for oedema around the eyes and nasal oedema. Each claw is individually scored for wall bulging, wall bleeding, sole reddening, detachment of sole from heel, reddening of heel, heel cracks, heel bleeding, detachment of heel, redness of coronary band, exudation from the coronary band, and clinical signs of necrosis in the coronary band (Figure 1A–D (middle bottom and bottom)). The total claw score should then be divided by 8 to give a score comparable to that of the other body parts. Field experience shows relatively good agreement between the findings of different claws. However, detailed data on this subject are not yet available. Additionally, congestions of the inner thigh veins can be recorded.

The different studies show some variation in the consideration of individual body parts and clinical variations. A precisely standardized procedure is not yet available. More studies are needed to define an optimal standard.

The resulting binary scores can be presented by the organ system as a percentage of the affected piglets. All recorded binary scores can be summed to form an organ score for the individual piglet, and all organ scores can be summed to form the total SINS score. To ensure equal weighting of the organ scores, they can first be *z*-transformed and then added together. Again, exact standardization has not yet been performed, because possible weighting functions for the individual organ scores cannot yet be reasonably estimated. In addition, it is likely that optimal weighting functions will differ in different herds. In any case, the SINS score corresponds to the normal distribution [24,25].

Lesions to tail, ears, skin, teats, and claws are generally highly prevalent in pig production systems [27,28,29]. Thus, other primary diseases must be considered differential diagnostically. This can be difficult under practical conditions, especially in older piglets and fatteners, where mixed forms of bites, mechanical irritation due to technopathies, infection, and SINS might occur [23]. Tail biting has particularly been identified in growing pigs [3,4,5,6,7]. Tail biting cannot be responsible for tail lesions in piglets at birth, and suckling piglets have never been reported to bite their tails and, especially, not the tail base, where lesions are common in SINS [21,22,23,24,25]. The literature does not describe how annular tail lesions could result from bites or technopathies, nor how technopathies could lead to inflammation and lesions in the tail base area. Studies that describe the occurrence of tail base lesions failed to discover any evidence of such external causes despite thorough monitoring of the piglets [21,22,23,24,25]. In addition, bites usually leave typical injuries that are easily diagnosable if the pigs are monitored and promptly bonitized.

Inflammation and necrosis of the ears of pigs are also commonly found [30,31,32,33,34]. For example, Pejsak et al. [33] described an ear necrosis syndrome in weaners and fatteners and discussed the possible association with (sub-) clinical infections with *Mycoplasma suis*, as well as *Staphylococcus hyicus* and *Streptococcus suis*. Park et al. [32] associated ear necrosis with environmental factors and infections with *Staphylococcus aureus* and *Staphylococcus hyicus*. In these previous studies, it remained, however, unclear whether the pathogens were causative or secondary invaders. Papatsiros [31] associated ear necrosis (porcine necrotic ear syndrome) in weaners with Porcine Circovirus Type 2 (PCV-2) infections and recommended to intensify vaccination control. Earlier, Pringle et al. [30] associated necrotic ear lesions with *Treponema socranskii*. In contrast, Weissenbacher-Lang et al. [34] described a comparable syndrome, denoted PENS (porcine ear necrosis syndrome), in 5 to 10-week-old pigs. The authors investigated the prevalence of infectious agents in the herd, such as Streptococci, Staphylococci, and Mycoplasmata, but could not detect any of these agents in the affected animals. The authors proposed that other (non-infectious) causes, such as mycotoxin exposure or stress factors, should be considered as primary causes of PENS. lndeed, recent investigations showed that the exposure of pregnant sows to mycotoxin DON (deoxynivalenol) resulted in typical necrotic tail lesions even in neonatal piglets [35].

Alopecia, swelling, coronary band injuries, and swelling and haemorrhaging into the claw corium were described in suckling piglets by Mouttotou et al. [36] and KilBride et al. [37]. The authors suppose that some of these lesions are associated with a reduction in suckling and active behaviours and a slower growth rate because of the pain associated with such injuries [38]. The authors attributed the alterations solely to mechanical irritation by the floor. Recently, however, clear evidence for an additional internal component in such lesions was provided by histopathological findings in piglets [23]. Even in newborn piglets [24], 20.5%, 65.1%, 76%, and 82.9% of the individuals were found with the swelling of heels, inflammation of coronary bands, redness of heels, and wall bleeding, respectively, directly at birth. An ongoing study in these piglets is disclosing the histopathological findings in the claws and ears (Wenisch, pers. communication) consistently with the findings at the tail base [24].

Several results provide evidence that the expression of individual clinical signs of SINS is clearly modified by environmental effects. Pigs with SINS react more sensitively to unfavourable barn floor conditions than those with healthy claws due to the primary load in the area of the heels, soles, and claws. Ears seem to be particularly affected by SINS in pigs with insufficient ability to regulate their body temperature [21]. This explains why the signs of SINS can vary significantly depending on the existing environmental factors in different herds and why the correlations between organ scores can be relatively low [26]. However, in a cohort of 646 piglets, none was completely free from signs of SINS, and of the seven body parts examined, including the tail base, tail tip, face, ears, teats, navel, and claws, 3.8 ± 1.07 of body parts (mean ± SD) were affected simultaneously in an individual [25]. In this study, forty percent of piglets were affected in at least five of the seven body parts.

## 4. SINS as an Endogenous Disease

Three main observations support the assumption that SINS is primarily an endogenous disease, even though it may be modified by technopathies and other mechanical stressors: (1) The simultaneous occurrence in such disparate body parts as the tail, teats, claws [22,24,25]; (2) evidence that SINS can be expressed before birth [24]; (3) evidence that inflammation originating from blood vessels can be present before birth when biting and mechanical irritation (e.g., from soil) are excluded and in piglets with (still) intact epidermis [23,24].

Clinical signs at the tail, claws, and ears were confirmed histopathologically. Vasculitis, thrombosis, intimal proliferation, oedema, and hyperaemia were detected together with intact epidermis [23]. Bristle loss is associated with inflammatory processes in the deeper parts of hair follicles [23,24]. Significant proportions of neonates may be affected. In the study by Kuehling et al. [24] on a conventional farm, 40–80% of neonatal piglets were affected by haemorrhages of the claw wall, coronal inflammation, redness of heels, bristle loss, and redness of the tail and ears. The inflammation could be characterized by granulocytes in considerable numbers, macrophages, and lymphocytes, indicating an onset of inflammation at least 4 days before birth [39], while the piglets were not older than 2 h. Thus, SINS must be assumed to have developed in utero.

Inflammation and necrosis have often been considered to result exclusively from biting and mechanical irritation. However, the above studies demonstrated that these cannot be the sole causes. Similar conclusions were already reached by Penny et al. [18]. The authors suspected that inflammations and necroses were the result of circulatory disturbances, which were triggered by vasoconstriction and aggravated by further circumstances. This hypothesis was confirmed by the above-mentioned findings of vasculitis, intimal proliferation, and thrombus formation at the base of the tail, directly cranial to the clinical lesions with inflammation and necrosis [24]. A second corroboration was the demonstration of a sharp drop in temperature from the affected base of the tail to the tip of the tail in piglets with SINS using an infrared thermography device [21]. In addition, several studies showed that inflammation of the tail tip (outside of biting events) is always associated with alterations at the tail base, while piglets with intact tail bases never show signs at the tail tip [23,24].

## 5. Hypothesis and Background of the Pathogenesis of SINS

**Figure 2:**
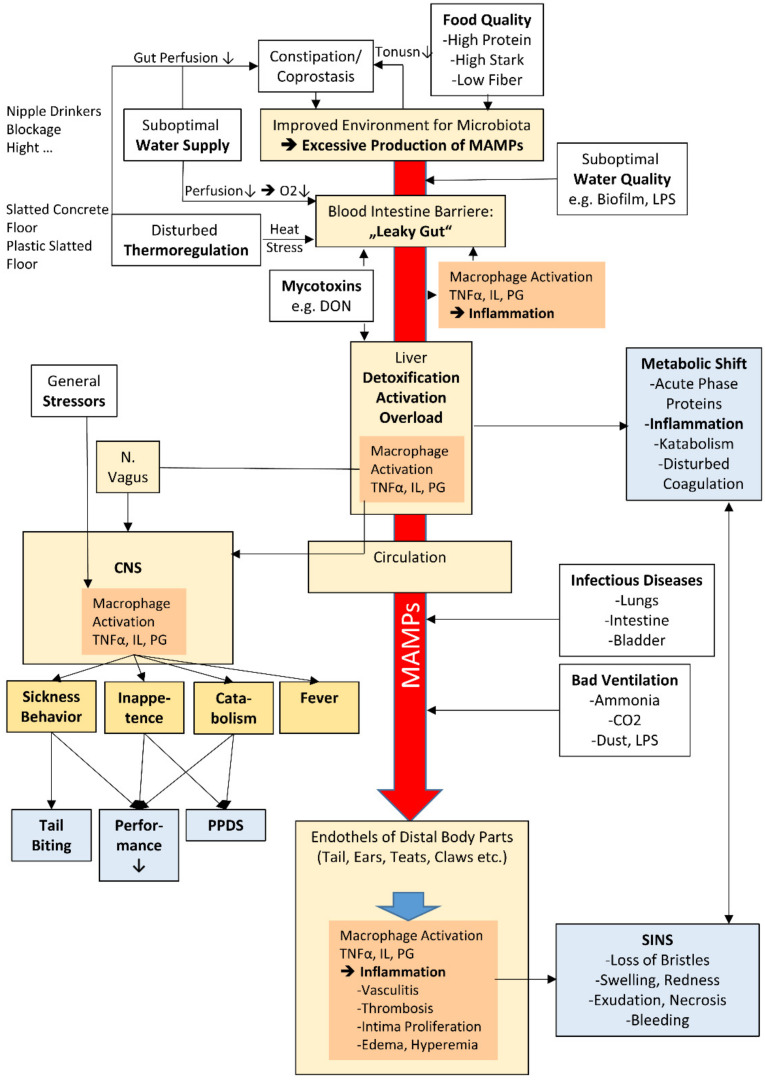
Hypothesis of the mechanisms leading to inflammation and necrosis syndrome (SINS). The focus is on microbe-associated molecular patterns (MAMPs, red arrow). Suboptimal water supply, thermoregulation, and food and water quality, together with mycotoxins, infectious diseases, poor ventilation, and other stressors (white boxes), increase the flooding of MAMPs (including LPS) at the levels of excessive proliferation of the gut microbiota and permeability of the blood intestinal barrier, liver, and circulation (light-yellow boxes). This triggers inflammatory responses at the intestine, liver, endothelia, and CNS (orange boxes). The consequences can be manifold. Among others, tail biting, reduced performance, PPDS in sows, metabolic shift, and SINS may develop. CNS: central nervous system; DON: deoxynivalenol; IL: interleukins; LPS; lipopolysaccharides; PG: prostaglandins; TNF: tumour necrosis factor.

Based on the histopathological findings and the data from infrared thermography, compiled in the previous paragraph, Reiner et al. [23], Ringseis et al. [26], and others suggested that SINS must be attributed to the local inflammatory processes in association with blood vessels. According to Van Limbergen et al. [35], necrotic tail lesions in newborn piglets can be associated to the exposure of the sow to deoxynivalenol (DON). Additionally, the necrosis of tails, ears, and coronary bands in suckling piglets might be directly associated with mycotoxins and LPS (lipopolysaccharides) from sows’ milk [34,35,40,41,42,43]. To date, no accurate dose-response studies are available that allow precise quantification of the relationship between ingested mycotoxin and LPS levels and clinical consequences in piglets and pigs of different ages and against a background of different mixing ratios and cofactors. Nevertheless, among others, mycotoxins and LPS could be directly or indirectly implicated in causing the inflammation of blood vessels in affected body regions. The following section collects findings that support this hypothesis and, thus, represent a possible starting point to explain the pathogenesis of SINS (Figure 2).

### 5.1. Microbe-Associated Molecular Patterns (MAMPs) from the Intestine

One of the most conclusive papers in this regard is that of Nordgreen et al. [44]. The authors suggested that problems in the microbiota, gut barrier, housing environment and hygiene, immune activation, mycotoxins, psychological stress, nutritional status, and feed composition can synergistically lead to inflammation directly or indirectly in association with LPS.

A major source of LPS and other microbial components with similar effects, collectively called microbe-associated molecular patterns (MAMPs), is the intestine (for a review, see reference [35]). Under physiological conditions, endotoxins always flood from the intestinal tract to the liver, where they are inactivated by Kupffer cells [45], whose clearance capacity can normally eliminate the endotoxins completely. Abnormally high levels of degradation products from the gut are observed in the presence of increased microbial proliferation, intestinal disease, a high protein-to-crude fibre ratio, and disruption of the blood–intestinal barrier [46]. They may also result from coprostasis that occurs as a consequence of fever, overfeeding, or an excessively high ambient temperature [47,48,49,50,51]. By overcoming the blood–intestinal barrier, MAMPs can reach the circulatory system. The blood–intestinal barrier is complex, oxygen-dependent, and sensitive to disruption. In pigs, damage to the blood–intestinal barrier directly results in increased LPS influx [47,48]. The blood–intestinal barrier in pigs is particularly susceptible to heat stress [49,50,51] and reduced gut perfusion with relative water deficiency and increased water requirements for thermoregulation [50,51] when contact cooling fails on dry concrete or plastic floors [52]. Mycotoxins (DON and similar substances) disintegrate the tight junctions of the blood–intestinal barrier and increase the LPS uptake in pigs [53,54]. They also directly cause intestinal and liver inflammation in pigs and develop synergistic effects with LPS [53,54,55,56,57]. By disrupting the blood–intestinal barrier, mycotoxins and LPS potentiate their mutual uptake.

Microbiota dysbiosis and intestinal barrier impairment are also associated with a number of chronic inflammatory disorders and systemic diseases in humans (for review, see references [58,59], and the pathogenic involvement of endotoxins is well-described [60,61,62].

### 5.2. MAMPs and the Liver

MAMPs are translocated from the gut to the liver via the portal vein. Since the liver acts as a barrier to degrade MAMPs due to the activity of macrophages (Kupffer cells) and other intrahepatic immune cells [63,64,65,66], the liver largely prevents MAMPs from entering the systemic circulation. However, in increased intestinal microbial proliferation, or if the intestinal barrier is disrupted (“leaky”), the liver is confronted with high concentrations of MAMPs. In this case, MAMPs are recognized not only by hepatic immune cells but, also, by hepatic parenchymal cells, which are abundantly equipped with specific MAMP recognition receptors, such as Toll-like receptors (TLR). Upon the recognition of MAMPs by TLRs, various inflammatory and stress signalling pathways, such as nuclear factor-kappa B (NF-κB), are activated. In addition, the c-JUN N-terminal kinase (JNK) and endoplasmic reticulum (ER) stress-induced unfolded protein response (UPR) are stimulated. This results in liver inflammation and impaired organ functionality [67]. First, the evidence for the induction of the inflammatory processes in the liver of piglets with SINS was provided by Ringseis et al. [26]. Proinflammatory genes and genes involved in the stress response, e.g., TNF, HP, ICAM1, SOD1, and CRP, were induced in piglets with SINS. This induction was accompanied by broad alterations of the metabolic pathways [26]. As a result of transcriptomic and metabolomic examinations, the metabolic processes dealing with lipid and fatty acid metabolism were shown to be particularly involved. This could be explained by the crosstalk between inflammatory signalling (NF-κB, UPR) and hepatic lipid metabolic pathways in inflammatory liver diseases [68,69,70,71].

### 5.3. Further Sources for MAMPs and Elevated Cytokines

Pathogens, particularly of the digestive tract and respiratory tract, are common sources of MAMPs and frequent causes of elevated cytokine levels in pigs (reviewed in [44]). Poor hygiene, dust, LPS, and high levels of ammonia in the air are regularly present in pig houses and lead to activation of the inflammatory cascade via the respiratory tract [72,73,74,75]. Pigs are exposed to massive stressors, including high housing density, lack of pen structure, regrouping, lack of opportunities to eat at the same time, disease, poor housing air, etc. [10,76,77]. A number of studies demonstrate that cytokine activation comparable to that elicited by MAMPs is induced in humans [78], rodents [79], and, also, in pigs [80,81,82,83].

### 5.4. Mode of Action of MAMPs, e.g., LPS

The pathogenicity of endotoxins in animals arises primarily from the release of endogenous mediators and is, thus, secondary [84]. The goal of this mechanism is to contain, control, and eliminate the bacteria. However, the excessive or systemic activation of mediators by endotoxins can result in severe disease and possibly even death as a consequence of the mediator effect. Target cells for endotoxins are neutrophils, macrophages, and platelets. Major mediators secreted in response to endotoxins include tumour necrosis factor-alpha (TNF-α), interleukin 1β (IL-1β), IL-6, and IL-8. This mode of activation was also confirmed in pigs as the trigger for post-partum dysgalactia syndrome (PPDS) [85,86].

IL-1 activates the vascular endothelia and lymphocytes. This leads to improved access for effector cells but, also, to tissue destruction. It also promotes the production of IL-6 and the development of a fever. IL-6 increases fever and stimulates the production of acute-phase proteins. The latter include, for example, C-reactive protein. They bind to bacteria and opsonize them or trigger a complement reaction. Elevated levels of acute-phase proteins were found in piglets with SINS [87]. IL-8 represents a chemotactic factor for leukocytes and improves the access of effector cells to the damaged tissue. TNF-α is the main trigger of inflammation (pain, redness, swelling, and increased temperature at the site of infection). In was elevated in the liver tissue from piglets with SINS [26]. Redness and warmth set in due to the increased vessel diameters, and swelling occurs due to increased permeability. The histopathological findings in piglets with SINS include hyperaemia and oedema [24]. Thus, there is an overall increase in the influx of immunoglobulins and complement proteins, as well as immune cells, and lymphatic drainage is promoted. Through the induction of adhesion molecules on the endothelia, monocytes and granulocytes are slowed down, docked, and infiltrated into the tissue (extravasation). The induction runs via the expression of selectin. These changes also result in increased blood clotting; small vessels are relocated, and pathogens are prevented from spreading further in the organism. Thrombosis in the tail base of piglets with SINS was found in the histopathological study by Kuehling et al. [24]. These essential properties of TNF-α to limit local inflammation can have disastrous consequences once an infection becomes systemic or LPS enters the blood in larger quantities. Systemic vasodilatation can then lead to shock and disseminated intravascular coagulation (DIC). In addition to the cytokines already mentioned, biogenic amines (e.g., histamine and serotonin), oxygen radicals (NO and H_2_O_2_), and, mediated by cyclooxygenase-2 (COX-2) activity, various arachidonic acid derivatives, especially PGE2 but, also, PGF2α, PGI2, and thromboxanes, are released [88].

The release of mediators occurs through activated defence cells (macrophages, Kupffer cells, etc.). Pathogens or their components invading via the intestine, urogenital tract, or fissures or wounds of the claws or skin are recognized by their MAMPs via specific receptors called Toll-like receptors (TLRs) in reference to the corresponding Toll receptors in *Drosophila*. LPS is primarily recognized by TLR-4 in several species [89,90]. To activate the TLRs, LPS must first bind to a lipopolysaccharide-binding protein (LBP). This complex, in turn, binds to CD14 receptors on the membrane of macrophages and is then coupled to TLR4 in an MD-2-dependent manner. The formation of this receptor complex activates a series of factors that introduce the signal that has arrived at the cell membrane into the cell and transfers it to the nucleus (overview in reference [91]). The final step is the formation of transcription factors—in particular, NF-κB, which starts their expression by binding in the promoter region of specific genes involved in inflammation, immune communication, and immunomodulation [46,92].

### 5.5. MAMPs and Consequences to the Periphery

The cytokines now expressed exert their effects locally in the tissues of the gut and liver. The binding of PGE2 to prostaglandin receptors controls the cyclo-AMP-mediated function of the ion channels of smooth muscle and peripheral and central neurons, thereby modulating their activity. This leads to a reduction in tone and slackening of smooth muscle of blood vessels and the intestine. A drop in blood pressure or stasis are possible consequences [93]. The permeability of the blood vessels is increased. Prostaglandins have an activating and potentiating effect on macrophages and the immune system. Increased pain sensitivity develops at the nerve endings of pain fibres, and afferent fibres of the vagus nerve are irritated. IL-1 and TNF-α act synergistically in this process. Excessive production of the mediators results in the following symptoms: general depression, fever, impaired motility of the smooth muscles of the intestine (up to stasis) and the urogenital tract, and leukopenia, followed by leucocytosis, metabolic disturbances, and disturbances of the circulatory system up to shock. In this context, the aforementioned symptomatology can be reproduced by endotoxins but, also, by TNF-α alone [88,94].

### 5.6. Central Effects of MAMPs

Through cytokines via the blood pathway [95] and through prostaglandins (PG) and cytokines via the vagus nerve [96], there is a reporting of inflammatory processes to the central nervous system [97,98]. This can lead to systemic reactions such as depression, anorexia, and fever. The flooding of LPS from the intestinal tract leads to activation of the complement system. Subsequently, C5 receptors on Kupffer cells in the liver are activated. Their stimulation induces the expression of cyclooxygenase-2 (COX-2) and, thus, the rapid formation of PGE2. PGE2 travels via the blood pathway to the preoptic anterior hypothalamus (POA), where it triggers a fever via prostaglandin receptors.

PGE2 action can also be directed to the POA by the stimulation of vagus terminals in the liver via the ventral noradrenergic bundle. The vagus signals are received by the medulla oblongata and neuronally transmitted to various brain centres. Activation of corresponding centres of the area praeoptica by noradrenaline causes an induction of COX-2 there and, thus, also PGE2-mediated initiation of a fever. Cytokines from the bloodstream can reach specific cytokine receptors in the area of the circumventricular organs, which do not have a blood–brain barrier. Here, they again trigger the formation of PGE2, which is the actual stimulus for the central effects. PG receptors in the paraventricular nucleus activate the hypothalamic–pituitary axis, those in the area praeoptica activate the fever centre, while disease-specific sensations and behaviours arise in the limbic system. Within minutes after the recognition of bacterial components in the organism by macrophages, fever is formed [99,100]. The purpose of fever is to promote the immune system and to inhibit numerous pathogens. The latter is realized both directly by the high temperature and by indirect effects, such as the lowering of various metal plasma levels required by bacteria at high temperatures. However, with increasing temperature, there are also more and more disruptions of important endogenous functions.

Disease sensations and disease-specific behaviour (sickness behaviour) eventually develop through the activation of PG receptors in the limbic system, with specific actions on ion channels [101]. The disease-specific sensation and behaviour that develop are not directly due to the fever but arise in parallel. These are highly organized behavioural changes after defence/immune stimulation, which are essential for dealing with pathogens, for example, in the sense of reserve conservation. The most important symptoms are dullness, reduced participation in the environment, secretion, sensitivity to pain, anorexia, and adipsia [88]. In this way, inflammatory processes are associated with mental illness in humans and rodents (reviewed in reference [44]). There is much to suggest that tail biting in pigs may also be influenced in this manner [44].

## 6. Genetic Effects

### 6.1. Effects of the Boar

Practical experience from pig farms with uniform sow base regularly shows evidence of boar or boar line effects on progeny SINS scores. Demonstrating a genetic basis of SINS would be an important milestone in combating the syndrome, as husbandry improvement measures, often insufficient on their own, could be supported by targeted selection of less sensitive boars. It would make control more effective and sustainable. This background led to a study with 19 boars (4 Duroc and 15 Pietrain boars) from 8 different breeding companies participating in the market in Germany [25]. The study was conducted on 39 sows, with each sow inseminated simultaneously with two different boars to increase the significance of the study. A total of 646 piglets were paternity tested and scored for signs of SINS on their third day of life. More than 70% of the piglets were affected at the tail base, ears, coronary bands, and heels. Bristle loss, swelling, redness, venous congestion, and claw wall bleeding occurred most frequently.

Offspring from Duroc boars had significantly lower SINS scores (4.87 ± 0.44) than offspring from Pietrain boars (10.13 ± 0.12). Within the Pietrain breed, SINS scores of offspring were significantly affected by the boar. Replacing the Pietrain boars by Duroc boars resulted in a 59% reduction in the SINS scores of their offspring under the given husbandry conditions. The cumulative percentage of three-day-old piglets from Duroc boars affected by SINS signs at the tail base was 43.5%. The corresponding values for the average Pietrain progeny were 165%. The progeny of the best Pietrain boars and the poorest Pietrain boars had values of 92.1 and 199.6, respectively. Exudation and necrosis occurred only in progeny of average (4.4%) and poor Pietrain boars (20.1%), but not in progeny of Duroc boars and best Pietrain boars. The effects on teats were even more pronounced.

Total SINS scores in the offspring of the best Pietrain boars were almost 40% lower than that of offspring in the poorest Pietrain boars. These findings confirm considerable genetic effects on the outcome of SINS under a given husbandry. The results clearly show that individual breeding companies had boars with both favourable and unfavourable distribution of SINS in their offspring. It has to be assumed that the expression of the SINS signs is significantly influenced by husbandry and feeding. It can be suspected that the absolute differences between boars might be weaker or stronger under more favourable or less favourable conditions.

However, the decisive results of the study were the clear differentiation between Duroc and Pietrain boars and the pronounced variation within the Pietrain boars that were included in the study. Such findings are currently being taken seriously by numerous breeding companies in various countries. Further studies are needed to characterize the genetic background of these effects and to make them useful to combat the syndrome.

### 6.2. Effects of the Sow

The genetics of the sow can also have significant effects on the SINS scores of the offspring [22]. Based on 20,000 pigs on 19 farms, it was found that more than 60% of fatteners from one of four sow lines (sows from four different relevant commercial breeding lines) had inflamed tails, while only 20–30% of fatteners from the other lines were affected. The same effect was observed for inflammation of the ears (40% vs. 0–13%). The fact that the sow line whose progeny showed high prevalence of tail and ear inflammation at the same time showed the least signs of biting, underlines the syndrome character of SINS on the one hand and proves on the other hand that tail lesions due to SINS or due to biting have to be strictly distinguished from each other. Of course, genetic differences a priori cannot be expected between all lines and populations.

## 7. Opportunities to Improve SINS by Environmental Options

Improving water and raw fibre supplies is generally accepted to have a positive impact on intestinal health and the improvement of animal welfare in swine [7,102,103,104]. Insufficient water uptake is a major risk factor for the development of postpartum dysgalactia syndrome (PPDS), a widespread disease in postpartum sows that is also related with inflammation triggered by MAMPs [105]. The resulting constipation is accompanied by exuberant bacterial growth in the intestine and flooding of endotoxins (MAMPs) [106,107]. This can be worsened by a lack of fibre [108,109], with the addition of fibre being “probably the most-cited factor to reduce PPDS” [110]. Constipation (coprostasis) is a leading sign and cause of PPDS [111,112,113]. Bacterial colonization of the endometrium, the bladder and the mamma were identified as further sources for MAMPs in PPDS [111,112,113,114,115]. Further resources for MAMPs were detected in injuries and fissures in teats and claws as well as laminitis in the sows [107]. Thus, sows with intact teats, claws, and skin, and which are free from coprostasis should have a lower burden on their piglets to develop SINS.

To test the role of water, fibre, and sow quality, including coprostasis, on the development of SINS in offspring, SINS scores for the offspring (120 suckling piglets, 120 weaners and 120 fatteners) from 40 sows were examined. Out of 123 sows, the 20 sows with the least alterations in teats, claws, and skin were contrasted to those 20 sows with the most severe alterations [23]. Sows were screened at day 50 of gestation. The offspring of 10 sows of good quality and 10 sows of poor quality were studied under standard housing conditions in a first run and under improved housing conditions in a second run, which included good crude fibre and sanitized water from open drinkers being continuously available to all ages. Coprostasis occurred exclusively under standard housing conditions, without improved water and fibre supply (R^2^ = 0.74), and more frequently in sows with poorer quality in skin, claws, and teats.

SINS scores in suckling piglets, weaners, and finishers of low-quality sows under standard housing conditions were highest but decreased significantly when housing conditions were improved. Sow quality had direct significant effects on inflammation and necrosis of suckling piglets and weaners under standard housing conditions. Offspring from sows with coprostasis had significantly higher SINS scores at every age.

Improved housing resulted in a 39, 56, and 81% decrease in SINS symptomatology in suckling piglets, weaners, and finishers, respectively. That the effects were detectable up to the fattening phase must be emphasized. The strongest effect of housing was the reduced incidence of coprostasis in sows. Thus, the husbandry effect could be split into a direct effect on the sows’ offspring and an indirect effect by provoking coprostasis in the sow. Husbandry improvements (fibre and water), sow quality, and coprostasis explained 57%, 67%, and 45% of the variance in SINS scores in suckling piglets, weaners, and finishers, respectively.

In conclusion, the use of fibre, sanitized water from open drinkers, and sows with healthy claws, healthy teats, and intact skin can have positive effects on the prevalence of SINS in suckling piglets, weaners, and fatteners. Avoiding coprostasis in the sow seems to play a particularly important role [23].

## 8. Conclusions

Focusing on biting is only one part of the solution to control tail lesions in swine and includes three major concerns: (1) Tail damage can be found without any action by other pigs and up to 75% of piglets can be affected. (2) The lesions are not limited to the tail. They can also be found in ears, heels, soles, claws, coronary bands, teats, navel, vulva, and face. (3) Environmental improvement alone often fails to overcome the problem.

Recognizing a primary endogenous syndrome as the cause of clinically detectable inflammation and necrosis at the aforementioned body parts leads to the second part of the solution: SINS. The syndrome can be triggered before birth and can be detected with considerable prevalence in neonates, suckling piglets, weaners, and fattening pigs.

The assumption that SINS is primarily an endogenous disease, even though it is modified by technopathies and other mechanical stressors, is supported by three findings: (1) The simultaneous occurrence of signs in disparate body parts such as the tail, teats, claws, and others; (2) the clinical expression of the syndrome before birth; and (3) pathohistological signs of vascular-associated inflammation with vasculitis and thrombosis, together with intact epidermis in newborn piglets, where biting and mechanical irritation (e.g., from the floor) can be excluded.

The idea of underlying circulatory disorders is supported by clinical and pathohistological results. A huge number of published findings support the hypothesis that these disorders might be due to microbe-associated molecular patterns (MAMPs), particularly from the intestine, that activate the defence cascade. The role of feed composition, nutrition, mycotoxins, gut microbiota, gut barrier, housing environment, hygiene, immune activation, and even psychological stress has recently been elaborated in detail. The resulting expectation of liver inflammation with massive switch of metabolism from anabolism to acute phase and inflammation was demonstrated.

Using sows with intact claws, mammaries, and skin and which are free from constipation can favourably and sustainably influence inflammation and necrosis in their offspring, from suckling piglets to finishing pigs. The use of crude fibre and sanitized water from open drinkers across all ages resulted in a further massive decrease in SINS signs at every age. Significant differences between offspring of Duroc and Pietrain boars and offspring of different Pietrain boars suggest the possibility of a sustainable improvement of SINS by breeding.

## Data Availability

Not applicable.
